# Automated Activity Tracking and Space Use Monitoring of Captive Jaguars with Machine Learning

**DOI:** 10.3390/ani16101504

**Published:** 2026-05-14

**Authors:** Laura Liv Nørgaard Larsen, Ninette Christensen, Trine Kristensen, Thea Loumand Faddersbøll, Anne Rikke Winther Lassen, Brian Rasmussen, Sussie Pagh, Cino Pertoldi

**Affiliations:** 1 Department of Chemistry and Bioscience, Aalborg University, Fredrik Bajers Vej 7H, 9220 Aalborg, Denmark; llnl23@student.aau.dk (L.L.N.L.); nchr23@student.aau.dk (N.C.); tkri23@student.aau.dk (T.K.); sup@bio.aau.dk (S.P.); 2 Aalborg Zoo, Mølleparkvej 63, 9000 Aalborg, Denmark; tlf@aalborgzoo.dk; 3 Randers Regnskov, Tropical Zoo, Tørvebryggen 11, 8900 Randers, Denmark; arw@regnskoven.dk (A.R.W.L.); bmr@regnskoven.dk (B.R.)

**Keywords:** activity budget, activity patterns, animal welfare, heatmaps, LabGym, *Panthera onca*

## Abstract

Monitoring both captive animals and wild populations is necessary to ensure adequate animal welfare and wildlife conservation. Existing monitoring tools, e.g., camera traps, enable surveillance, yet analysis can prove time-consuming and labor-intensive if handled manually. The automated nature of machine learning (ML) reduces observer bias and manual workload and improves assessment capacity of behavioral monitoring tools that are often used by staff at zoological institutions. This study investigated the activity and space use of three captive jaguars (*Panthera onca*) through automated individual recognition, activity tracking, and heatmap visualization using an ML model trained on video footage. In total, 123.8 h of video footage was recorded of the jaguar enclosure in Randers Regnskov, Tropical Zoo. The ML model analyzed all videos containing jaguars from one day. The model achieved satisfactory performance based on its evaluation metrics (mean average precision, recall, precision, and F1-score). The ML model showed repeated movement tracks within specific enclosure areas. The jaguars exhibited significantly more inactive than active behavior and did not seem to exhibit natural bimodal nocturnal or crepuscular hunter activity patterns. It should be stated that, due to the small sample size of only three jaguars and 24 analyzed hours, this study is a proof-of-concept to demonstrate the potential of ML methods as valuable tools for individual recognition, activity tracking, and monitoring of space use to aid in future animal welfare monitoring.

## Simple Summary

Animal welfare can be estimated by assessing the behavior of captive animals through the lens of a behavioral baseline from wild conspecifics. However, manual assessments of animal behavior can be time-consuming and prone to bias. In this study, a machine learning model was trained with LabGym on footage of three captive jaguars to automatically detect and track their activity and inactivity. Additionally, heatmaps for visualizing space use were created based on the automatic tracking of their positioning. The machine learning model showed promising results based on its evaluation metrics. The model indicated repeated movement tracks within certain areas of their enclosure. The jaguars exhibited significantly more inactive behavior on the analyzed day and did not seem to exhibit natural bimodal nocturnal or crepuscular hunter activity patterns. Given the small sample size of only three jaguars and an analyzed period of only 24 h, this study remains a proof-of-concept, yet it still suggests that machine learning methods appear to be a promising tool for examining activity and space use of jaguars. Therefore, this approach might be valuable for saving time and reducing bias, which may lead to enhanced information flow, thus improving animal welfare and wildlife conservation.

## 1. Introduction

### 1.1. Ecology and Conservation

The jaguar (*Panthera onca* Linnaeus, 1758) is the largest predator in its natural home ranges, spanning from northern Mexico to northern Argentina, where it mostly lives in tropical or subtropical forests [1,2,3,4,5]. The jaguar is listed as near threatened on IUCN’s Red List, with a generally decreasing population [6]. Moreover, local populations of jaguars are critically threatened [5]. Its historic range has been reduced by about 50% in the last century, mainly due to habitat loss and fragmentation caused by anthropogenic actions. High food requirements and wide-ranging behavior combined with prey depletion elicit conflicts with humans and livestock, which may result in persecution of the jaguar [5,7,8,9,10]. The loss of jaguars in an area may have significant consequences with cascading effects on the local nature [8].

Conservation of jaguars is essential for maintaining ecosystem integrity and biodiversity within their habitats, thereby safeguarding numerous co-occurring species [8,11,12]. By understanding the behavior and way of life of jaguars, it becomes attainable to manage their populations [13,14] and to predict how disturbances, such as habitat loss and fragmentation, might influence them [15,16,17,18]. Studying the behavior and welfare of wild felines may be a challenge due to difficulty in localizing them in dense forests, low population densities, or avoidance of humans [3,15,19]. Camera traps and GPS technology have shown to be valuable in examining Neotropical felids, such as jaguars, for evaluating their population sizes, densities, and movement ecology [3,20,21,22,23,24]. However, in captive settings it is easier to study movement and cognition and how animals respond and adapt to different situations, as it offers greater experimental control and more detailed observations [25].

### 1.2. Welfare of Jaguars in Captivity

Animal welfare is about the well-being of animals [26], and it is an important ethical aspect of managing animals in captivity and monitoring wild animals for conservation purposes [19,26,27,28,29,30]. Animal welfare often depends on the naturalness of their living conditions, meaning that welfare is assessed using a wild conspecific behavioral baseline [19,26,27,29,30,31]. For example, studies on behaviors in wild jaguars concluded that jaguars are solitary and have large home ranges with sex-specific variation, with males having a tendency toward larger ranges and to travel larger distances per day than females [10,32,33,34,35]. To achieve high welfare in captive animals, it is not necessarily better to fully mimic wild conditions but instead provide positive natural aspects and withhold some natural stressors like violent fights, as they are undesirable in captive settings [26,29,30]. For example, conventionally solitary animals, such as jaguars, may benefit from being part of a group in captive conditions, as it could heighten the social complexity, increase stimuli, enable positive social behaviors, and reduce the risk of stereotypies [27,29,30,36,37]. Collaborative behavior through potentially long-lasting coalitions among male jaguars and other social interactions between both sexes have been observed in the wild, suggesting that sociality may be more common in jaguars than previously thought [38,39].

If jaguars in captivity are unable to express basic wild behaviors, e.g., walk, climb, and swim, they can become stressed and frustrated, which can lead to aggression and stereotypic behaviors such as pacing [27,29,30,31,40]. Another negative aspect of captivity for jaguars is increased inactivity both day and night compared to their wild conspecifics, which, when excessive, might result from lethargy, disease, or a stressful situation with low animal welfare [26,31,41,42]. To increase animal welfare, the implementation of enrichment, which promotes choice and sensory stimulation (e.g., through balls, scent trails, and cardboard boxes containing food), as well as increased enclosure size and complexity can be an effective way to reduce the amount of time spent on stereotypical behaviors and inactivity. Instead, more time is spent on positive and activity-related behaviors and is thus a contributing factor to strengthening the welfare of the animals [27,29,30,43].

### 1.3. Activity Budgets

Activity budgets are used for examining the behavioral patterns of animals to determine if they spend an excessive, normal, or inadequate amount of time on each behavior [26,31,35,44,45,46]. Activity budgets are also used for examining daily behavioral activity patterns throughout the 24 h of the day. Hence, activity budgets are also an opportunity to compare the behavioral pattern of captive animals with that of wild animals to determine their naturalness, which can be a part of the welfare state [26,31,44,45,46,47]. For example, wild jaguars have different movement patterns and activity levels throughout 24 h, depending on their age, sex, and reproductive status, with the average being 11.7 h of activity per day [35]. Furthermore, jaguars are mostly nocturnal and crepuscular, but activity patterns vary between individuals [32,35,41,48]. Activity budgets can be used as a part of assessing welfare through a behavioral assessment by knowing when and how much time is spent on positive and negative behaviors. In this regard, it can be important to know the amount of time spent on stereotypies and inactivity, and at what time of day these are expressed [26,31,44,45,46,47]. However, there is uncertainty in deciding whether an expressed behavior is positive or negative [31]. For example, inactivity can be because the animal is content and resting, or it can be due to boredom, or it might be indicative of lethargy if excessive and associated with low behavioral diversity [31,42,49]. Furthermore, some behaviors, such as aggressive interactions, may have very negative effects on welfare, even if they are brief [31]. Although it can be difficult to assess the negativity of aggressive behaviors, as they can be part of maintaining a social hierarchy or other social interactions, which might be positive [50,51]. However, animal welfare cannot be evaluated only with a behavioral assessment; it should be assessed with a holistic approach that includes the parameters of the Five Domains (nutrition, environment, health, behavior, and mental state) [52]. Still, monitoring an animal’s behavior is an important aspect of assessing its welfare.

### 1.4. Machine Learning

Behavioral assessments and space use analyses in zoological institutions can be completed in different ways, either by manually observing the animals in their enclosures or by applying videography with human observations and judgments [53]. It can be feasible to complete such manual analyses on jaguars, as seen in other studies [41,43,54,55], but it is highly time-consuming and can potentially lead to unforeseen problems regarding observer bias, subjectivity, or an observer’s inability to properly assess a behavior [53]. Additional challenges may also arise when observing multiple individuals simultaneously [56]. Some of these logistical problems might be ameliorated with the usage of emerging techniques, such as machine learning (ML), as it offers a more objective and standardized way to analyze behaviors [53]. The benefit of ML is its adaptability and trainability, enabling it to handle some of these intractable or labor-intensive tasks in a shorter amount of time [52]. An ML model can be trained to automatically identify, track, and categorize an animal’s behavior and therefore aid in behavioral analysis and assessments [53,57,58]. These models work by first identifying and outlining an animal in video data, after which any spatiotemporal changes to the outline will enable the model to infer an expressed behavior [59]. To our knowledge, there are no studies to date that have investigated the use of ML methods on footage of jaguars for behavioral and space use analysis. Although utilization of ML models may prove valuable, they are not without difficulties. For example, ML techniques may require some amount of coding knowledge. Here, more user-friendly graphical user interfaces (GUI), as seen in LabGym, may prove more accessible than programs that require a higher level of coding knowledge [59]. User-friendly ML options to supplement the manual observations obtained by zoo staff may be needed, if ML methods are to be used widely as a part of animal welfare monitoring in zoological institutions [60]. In this regard, LabGym is an ML program that keeps the entry level for development and the application of automated behavioral and space use analysis low [59].

The aim of this proof-of-concept study is to investigate the automated individual recognition, activity tracking, and space use monitoring of captive jaguars by applying a user-friendly ML program, as it could aid in future animal welfare monitoring by limiting observer bias and reducing time requirements.

## 2. Materials and Methods

### 2.1. Data Collection, Animals, and Enclosure

Footage was acquired by filming the jaguar enclosure in Randers Regnskov, Tropical Zoo (“Randers Rainforest, Tropical Zoo”) (Randers Regnskov) in Denmark. In this study, the activity and space use of three captive jaguars were analyzed. These were Chica, the melanistic female, Bentor, the male, and Sibba, their cub (Table 1). Their diet consists of varied meats, for example, deer or goat. They are fed 17 kg in total outside every Tuesday at 13:30 and Saturday at 13:00. At feeding time, the male jaguar is separated from the female and the cub as a precaution to avoid potential fighting, as jaguars are naturally solitary and food dominant.

**Figure A1:**
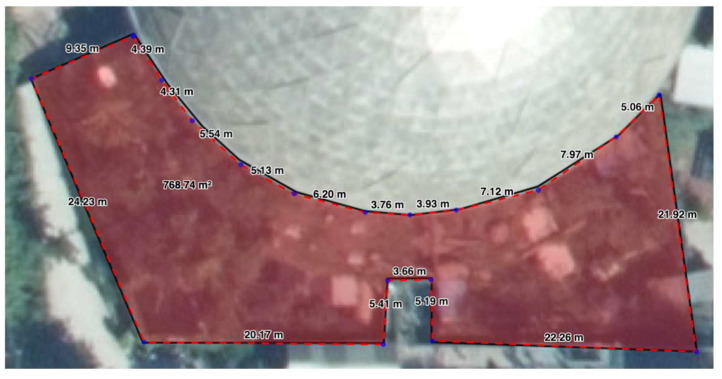
Aerial photo of the outdoor area of the jaguar enclosure in Randers Regnskov, Tropical Zoo (red area), with measurements of area (m^2^) and distances (m).

**Figure A2:**
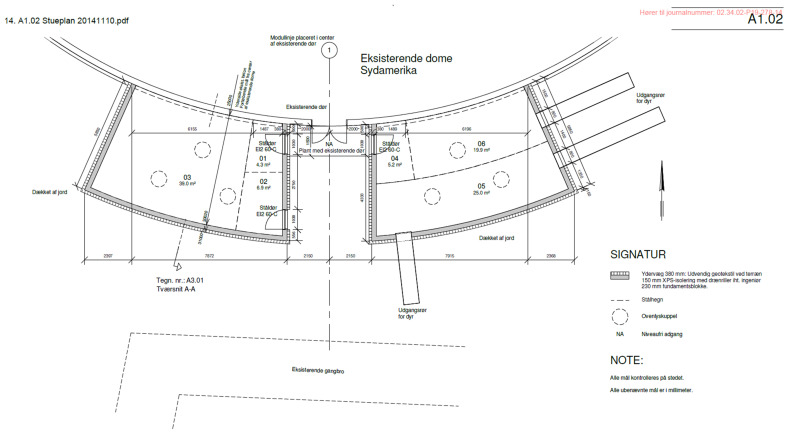
Floorplan of the indoor area of the jaguar enclosure in Randers Regnskov, Tropical Zoo, to the right. Unspecified measurements are given in millimeters.

The enclosure is separated into two inner and two outer areas. The two areas of the outdoor enclosure total about 769 m^2^ (Figure A1). The outdoor enclosure includes varying amounts of foliage, a tin roof atop a hilltop for sunbathing, a brook, and a small pool that allows for swimming. The inner stable enclosure totals 49.9 m^2^ (Figure A2) and includes an open wooden crate in the middle of the room, a bunk bed, cabinet, chest, and a big glass window. Lastly, there is also a backstage area not visible to the public. Unless there was a need to close off certain parts of their enclosure for management purposes, e.g., for feeding or cleaning, the jaguars could move freely between all areas of the enclosure–both day and night.

**Figure 1:**
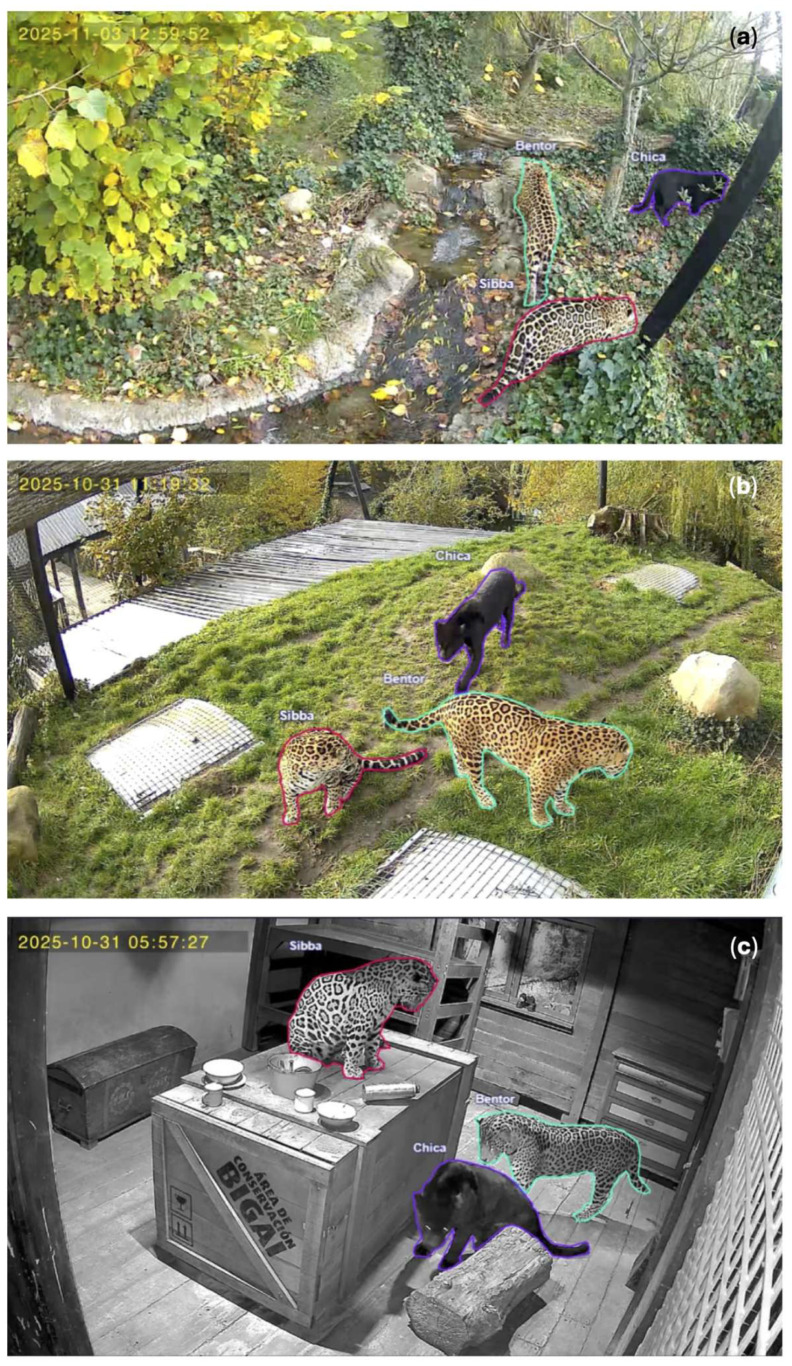
The three camera angles that were used to film the enclosure in Randers Regnskov, Tropical Zoo. The captive jaguars were annotated as ‘Bentor’ (turquoise), ‘Chica’ (purple), and ‘Sibba’ (pink) in Roboflow. The letters in the corners indicate the camera angles: (**a**) Angle from camera a outdoors, pointing into vegetation with a path and a small brook; (**b**) angle from camera b outdoors, pointing at a roof and a flat grassy area that functions as a connection point; (**c**) angle from camera c indoors in the jaguar stable.

The jaguars were video recorded continuously with three cameras (Hunters Choice LPV Live Panorama Vildtkamera, Guntex A/S, 6900 Skjern, Denmark) at 15 FPS, with a frame size of 1296 × 2304 pixels and with an FOV of 90 degrees for six days in 2025 from 30 October 11:25 to 4 November 15:18. Continuous focal sampling was chosen, as it allows for more detailed data collection [61]. The high resolution was needed to be able to better recognize the distinct pelage patterns of the jaguars, especially if they were further away, moving, or partially obscured. We chose the camera positions in collaboration with the zoo staff to have them pointed at locations where the jaguars tended to spend a lot of time or used for moving around their enclosure. This would ensure as little occlusion as possible for such a large enclosure with potentially many blind angles. However, large areas of the enclosure remained out of view, as the cameras covered approximately 30.3% of the enclosure. One camera was placed indoors in the jaguar stable, two cameras were outside, one pointing at a roof that the jaguars like to relax on and a flat grassy area that functions as a connection point between the two outside areas of the enclosure and the other pointing into vegetation with a path and a small brook (Figure 1).

An important aspect of filming continuously was minimizing the disturbance of the jaguars. During the process of setting up the cameras, the jaguars were separated into another part of their enclosure. This separation and relocation occur often and are therefore not thought to disturb them. The jaguars showed no reaction to the cameras, and their normal routines were not changed or impacted by this study. All proceedings regarding the jaguars were in accordance with the zookeepers to not impact the animals’ welfare or to stray from their normal routines.

During the filming period, there was a mix of rainy, cloudy, and sunny weather, and the temperature ranged from 11 to 13 °C in the daytime and from 3 to 10 °C at nighttime. The video recordings of the captive jaguars resulted in 123.8 h of video that were automatically split into shorter videos of about 2–3 min on average by the cameras. The jaguars were only visible in around 24.2% of the videos recorded.

We trained the ML model in the categorizations described in the ethogram that was developed for this study based on prior manual behavioral observations (Table 2). As the ML model struggled with classifying more advanced behaviors, the methodological decision was made to instead create two categorizations to examine activity and inactivity as a proof of concept, due to limited training data. The model was then used to analyze activity and inactivity through continuous focal sampling and plot coordinates of the centers of the individuals for the creation of heatmaps. Both behavioral categories were treated as states [62].

### 2.2. Development of Machine Learning Model

**Figure 2:**
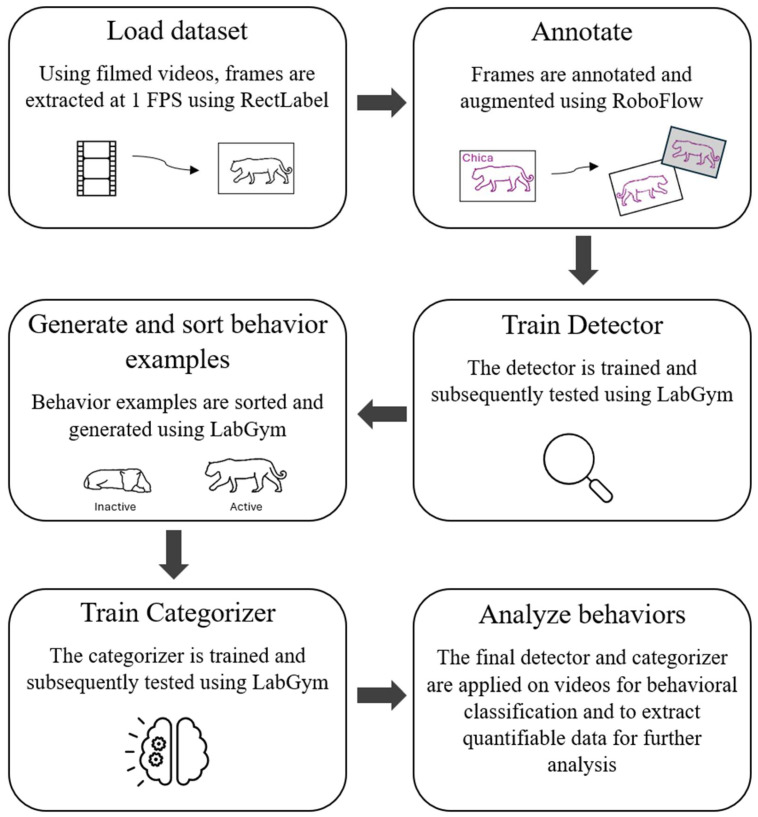
Illustrative pipeline used in this study for training machine learning model to both detect jaguars and categorize their behaviors [66].

LabGym (Version 2.9.6) [59] was used to create the detector and categorizer for behavioral and space use monitoring (Figure 2). LabGym is a computational tool using ML with a deep neural network (DNN) and a holistic approach to assess both “pattern image” (an animal’s motion pattern) and “animation” (an animal’s spatiotemporal details of a behavior) from video footage. These elements, coupled with quantitative measurements and thorough training, enable the accurate recognition of behaviors across a wide range of species [59]. It is paramount that thorough and diverse training of both the detector and categorizer is done to assure accurate predictions even amidst changing environments, camera shifts, occlusions, etc., to maintain the model’s precision [63,64]. For the creation of the detector and categorizer, LabGym’s Extended User Guide [65] was used for the guidelines.

Initially, and in order to reduce redundancy between frames, a dataset was created by extracting frames at 1 FPS from videos of the jaguars using the program RectLabel (Version 2025.12.17) [67]. We then manually annotated these frames in Roboflow using “Instance segmentation”. The entire outline of a jaguar was annotated with polygon segmentation using both automatic smart select and manual polygon tool [68]. Each frame was thoroughly reviewed multiple times to make sure no erroneous annotations would be used to train a detector.

We trained a detector to perform individual recognition by annotating each jaguar present in a frame as either ‘Bentor’, ‘Chica’, or ‘Sibba’ (Figure 1). As jaguars have individually recognizable pelage patterns, individual recognition would be possible [20,69]. It was important that a sufficient number of individual and varied frames were included, so that the detector would be able to spot and correctly identify jaguars in unseen videos. It was also important to show varied angles and frames that would include different behaviors. Frames without jaguars were also included in the training and testing images for the detector. Frames containing a bisected silhouette of a jaguar were excluded from annotation for the detector due to the outline consisting of multiple polygons, which may cause erroneous detections. Of the total annotated images, 3091 images were used for training the detector (80%), and the last 773 images (20%) were used for testing purposes (Table 3a). Bentor was annotated 1223 times, Chica 1879 times, and Sibba 1613 times. The training frames underwent additional augmentation using flip (horizontal and vertical), blur (up to 2.5 px), noise (up to 0.1% of pixels), and brightness (15% darker and 15% lighter), and the number of training frames was scaled up 5× to create a dataset version with 15,455 images. These augmentations and their intensities were chosen, as they either represent potential problems that the ML model may face when detecting the jaguars or could prove beneficial for training the detectors’ rigidity. For example, blurring, changing brightness, or noise may occur in the footage when it rains, gets foggy, the luminosity changes throughout the day, or when the lights are turned on or off.

This dataset version was then used to train the detector in LabGym with an inferencing frame size of 1296 pixels and 20,000 iterations on a laptop with an Intel Core i7-9750H CPU, NVIDIA GeForce RTX 2060 GPU, Windows 11 operating system, and Python version 3.10.11. The training took around three hours and resulted in a mean average precision (mAP) of 83.96% for the detector, when tested on the testing frames of the captive jaguars (Table 3a).

When training the categorizer to recognize behaviors, the videos were first pre-processed using LabGym to only contain footage with visible jaguars. Next, behavior examples and pattern images were generated for each individual as 1 s videos (15 frames) that were sorted based on the ethogram (Table 2). Behavior examples with erroneous detections were discarded and not used to train the categorizer. The resulting behavior examples and pattern images were reduced to a size of 512 × 512 pixels to generate 1157 ‘active’ and 943 ‘inactive’ training examples for training the categorizer (Table 3b).

Lastly, an “Interact advanced” type categorizer was created to recognize the sorted behaviors with the highest network complexity of 7 for both the animation analyzer level and the pattern recognizer level. The input shape was chosen to be 32 for both the animation analyzer and the pattern recognizer. Fifteen frames were used for animation and pattern image. The background and body parts were included. An interaction distance of 0 and a standard deviation of 25 were chosen. Finally, the training examples and validation data were augmented by LabGym using the default settings on a laptop with a Windows 11 operating system, AMD Ryzen 7 5800H with Radeon Graphics CPU, NVIDIA GeForce RTX 3070 Laptop GPU, and Python version 3.10.11. The augmentation process took approximately 5 h. The same laptop was used for training the categorizer, which took approximately 9 h. The categorizer was subsequently tested and achieved a satisfactory precision, recall, and F1-score of 0.92 for active behavior and 0.90 for inactive behavior when tested on 2100 sorted behavior examples of the jaguars (Table 3b).

### 2.3. Use of Machine Learning Model

Firstly, a day’s worth of videos from the jaguar habitat in Randers Regnskov was analyzed from the day with the most evenly distributed number of videos across the cameras. For the analysis, the frame size was reduced to 288 × 512 pixels, and the batch size was set to 1 to prevent memory errors. For the categorizer, the uncertainty level was set to 10%, and there was no minimum length for behaviors. Beforehand, the beginning time for analysis in each video’s file name was specified to limit the amount of video without jaguars visible. In total, 326 videos out of 429 videos from 1 November 2025, with jaguars visible, were successfully analyzed. A NoneType area error message from LabGym was received on the remaining 103 videos, possibly because the detector did not detect the specified individuals and could therefore not calculate an individual’s area. All quantitative measurements were analyzed with normalized distances [65] as well as the observed behaviors.

Several files with data from LabGym were given for each successfully analyzed video. These were consolidated into large datasheets using RStudio (RStudio version 2026.01.1 and R version 4.3.2) [70] to be used in the final data analysis. For behavioral analysis, all behaviors with less than 80% probability were removed from the dataset when analyzing the behaviors. In contrast, all data from the analyzed videos were used for the analysis of coordinate positions. The duration of each instance of a behavior was calculated from the start time of the current behavior to the start time of the next behavior instance. The hour of day that the video took place was also noted in all datasets. The coordinates, hour of day, and behavioral data were used for constructing plots for data analysis.

### 2.4. Activity and Space Use Analysis

To examine the activity, inactivity, and space use of the jaguars in Randers Regnskov, several plots were constructed using either Excel (Version 2604) or RStudio based on the data from the analyzed videos. The following packages were used for data analysis within RStudio: dplyr [71], ggplot2 [72], purrr [73], tidyverse [74], stringr [75], and writexl [76]. ChatGPT was occasionally utilized to aid in R code development [77]. Firstly, three heatmaps were constructed from the coordinates to show the space use from the point of view of each camera angle in Randers Regnskov. These were visualized using the 3D-map function in Excel [78]. Secondly, three summarized activity budgets were constructed as bar charts-one for each jaguar in Randers Regnskov. These show the amount of time in percentage that each jaguar spent on active and inactive behavior. Additionally, chi-square tests were used to ascertain whether the difference between time spent on activity and inactivity was significant for each jaguar. Next, two activity budgets were constructed as activity curves, showing the expression of active and inactive behavior throughout the 24 h to estimate the combined circadian rhythm of the three jaguars. The background of the diagrams is colored grey and white to indicate night and day periods, respectively. We chose to combine the behaviors of the jaguars, because there was not much behavioral data from each individual for every hour of day. On average, 9020.2 s of behavior from throughout the 24 h of the day were determined for each jaguar. For the remaining time, the jaguars were either not visible or exhibited behavior with an inadequate probability to use for further behavioral analysis.

## 3. Results

### 3.1. Space Use

**Figure 3:**
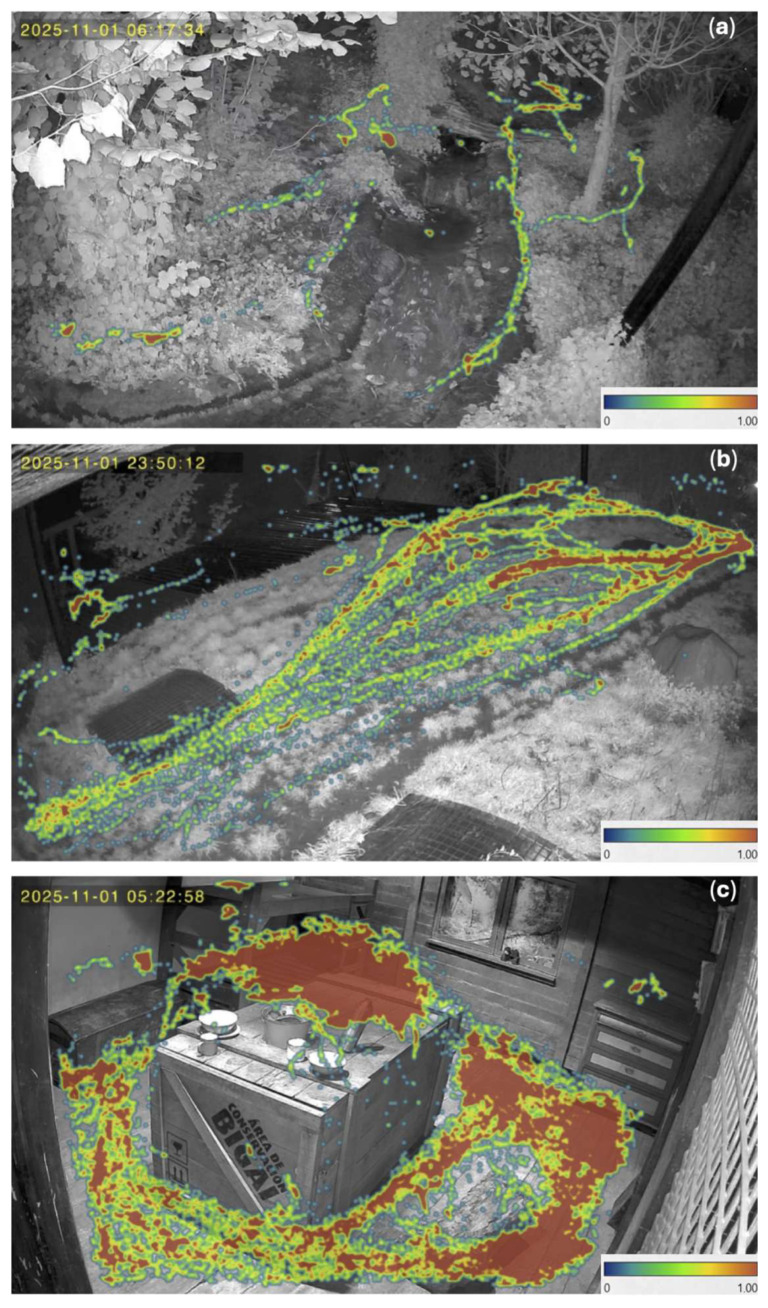
Coordinates of the centers of the jaguars (*n* = 3) in Randers Regnskov, Tropical Zoo, combined from all analyzed videos from the analyzed day, depicted on corresponding screenshots of the camera angles. The legends at the bottom indicate detection intensities, with 0 being low and 1.00 being high. The letters in the corners indicate the camera angles: (**a**) angle from camera a outdoors, pointing into vegetation with a path and a small brook; (**b**) angle from camera b outdoors, pointing at a roof and a flat grassy area that functions as a connection point; (**c**) angle from camera c indoors in the jaguar stable.

The heatmaps showed clear movement tracks, especially outside, and general space use preferences for the jaguars (Figure 3). In camera angle a, clear tracks are seen alongside the brook and in the vegetation to the left and right (Figure 3a). Camera angle b (Figure 3b) shows a connection point between the outside areas of the enclosure. There are clear tracks on the hill from when the jaguars move between the outside area with the brook to the right and the other outside area that is out of sight to the left. Camera angle c (Figure 3c) shows the indoor stable with space use focused on and around the crate in the middle of the room.

### 3.2. Total Expression of Activity and Inactivity

**Figure 4:**
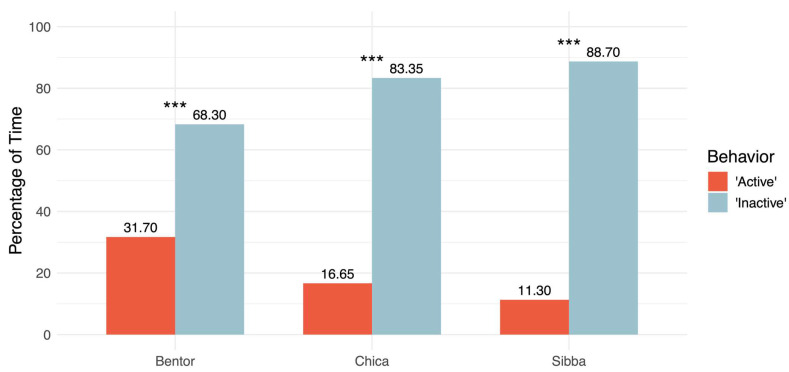
Distributions of percentages of time spent on active (red) and inactive (blue) behavior on the analyzed day for each jaguar (*n* = 3) in Randers Regnskov, Tropical Zoo, with bars depicting the percentage of observed activity and inactivity. The *x*-axis shows the different jaguars: Bentor, the adult male, Chica, the adult female, and Sibba, the female cub. The *y*-axis shows the percentage of time used on activity and inactivity. The number above each bar is the percentage of time used on activity and inactivity. The asterisks indicate significances from chi-square tests on the individual distributions of active and inactive behavior.

All jaguars spent a significant majority of the observed time inactive (*p* < 0.001), ranging from 68.3% in Bentor, the adult male, to 88.7% in Sibba, the female cub (Figure 4).

### 3.3. Expression of Activity and Inactivity Throughout 24 h

**Figure 5:**
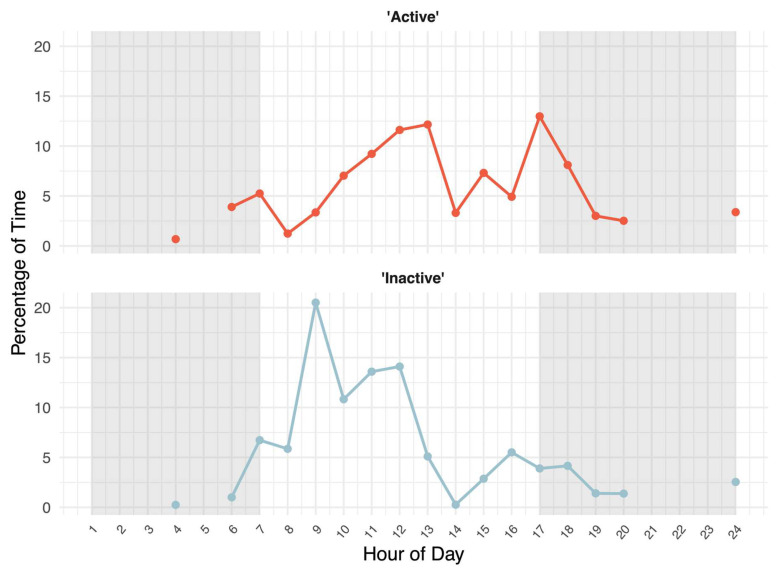
The activity curves of the two 24 h behavioral activity patterns from the analyzed day, combined for the jaguars (*n* = 3) in Randers Regnskov, Tropical Zoo. The *x*-axis shows the hour of day. The background of the diagrams is colored grey and white to indicate night and day periods, respectively. The *y*-axis shows the percentage of time spent on active (red) and inactive (blue) behavior within each hour of day. Missing lines of the curves indicate when there was no footage of jaguars within that hour of the day.

The three jaguars were observed to be the most active around noon (11th to 13th hour) and less in the morning and night (Figure 5). The jaguars showed the most inactivity between the 9th and 12th hours with a more even distribution for the rest of the day. No behavior was observed from the first to third hours, in the fifth hour, or from the twenty-first to twenty-third hours due to lack of footage, which is indicated by the missing lines of the curves in these intervals (Figure 5).

## 4. Discussion

### 4.1. Space Use Assessment

When constructing heatmaps to automatically examine the space use for the jaguars in the three different camera angles, a clear pattern appeared (Figure 3). The jaguars’ movement pattern appeared clustered in the indoor stable (Figure 3c). These clusters, on the crate in the middle and on the floor to the right, coincide with areas where the jaguars were often manually observed to rest and sleep. Furthermore, the jaguars’ movement patterns appeared repeated, streaky, and deliberate in the outdoor enclosure (Figure 3a,b). For example, it seems that the jaguars have been moving along the brook and within the vegetation (Figure 3a). Moreover, it seems that the jaguars were especially using the grassy area on the hill for traversing from one side of the enclosure to the other (Figure 3b). In addition, it seems that the jaguars have been avoiding walking on the roof top window as seen by the lack of detections that forms a circle to the right (Figure 3b). Here, the use of ML highlights distinct tracks throughout their outdoor enclosure, which may suggest possible locations for patrolling routes. Although it must be stated that, due to the methodological approach used in this proof-of-concept study, which relied on broader behavioral categories (active and inactive behavior), direct analysis of patrolling behavior would not be possible. Territory patrolling is seen in wild conspecifics, where both sexes of jaguars tend to patrol and guard their territory [35]. Because it is important for animal welfare that a captive animal exhibits positive aspects of wild behaviors [26,29,30], similar camera setups could perhaps be established long-term to further examine the possible locations for patrolling routes of captive jaguars or other species with a similar patrolling tendency. However, repeated movement tracks could perhaps also suggest stereotypical behavior, which might be indicative of a poor welfare state [26,31]. Difficulties arise when studying these invariant repetitive behaviors, as they can be expressed over a longer time scale. Therefore, training an ML model to analyze stereotypical movement behavior may require a longer window for behavioral scoring [79]. However, distinguishing between general locomotion and stereotypic movement, i.e., normal and abnormal behavior, might be challenging, as repeated general use of certain paths may be misinterpreted as stereotypic movement when examining space use using heatmaps [80].

Additionally, knowing an animal’s space use in an enclosure may also aid in optimizing the animal’s enclosure usage. For example, a study of pygmy hippopotamuses (*Choeropsis liberiensis*) showed promise with using olfactory enrichment to alter and increase the captive pygmy hippopotamuses’ space use [56]. Other studies have also demonstrated that computer vision technology can be an asset in examining space use, position tracking, and behavioral analysis to help reduce labor-intensive manual observations [53,57,63,81].

### 4.2. Activity Assessment

Daily activity budgets constitute an important aspect of behavioral welfare assessments and have been constructed for both captive and wild animals [26,31,35,44,45,46]. The results suggest that the male, Bentor, expressed more active behavior than the reproductive female, Chica, and the female cub, Sibba (Figure 4). Similar results on wild jaguars have been concluded in other studies that observed males to move longer distances per day than females [10,34]. However, the results are partly in contrast to a study by Jędrzejewski et al. (2021) [35] on wild jaguars that observed reproductive female jaguars, which have to care for their cubs, mate, and defend territory, to have had the longest activity time. They also observed that males had the second-longest daily activity, and cubs had the least. A reason for the difference between this proof-of-concept study and their study might be that the small sample size of three jaguars in this study was recorded in a captive environment and only for a single day. Therefore, if a different day or multiple days had been examined, the results of which jaguar was the most and the least active might have been different.

Inactivity is common in species of wild felids in zoos and has been found to be excessively expressed both day and night despite them being nocturnal animals [41,44]. All jaguars in this study spent a significantly larger amount of time being inactive than active (Figure 4). Moreover, a possible increase in inactivity was observed during the day, from the 9th to 12th hour (Figure 5). Furthermore, the jaguars seemed to be sleeping or resting at night, as it was observed that the three jaguars presumably woke up, stretched, and exited the crate in the indoor stable at the start of the fourth hour (around 3:00). The lack of footage of the jaguars at nighttime could therefore be explained by them being out of sight, for example in the crate or outside visible angles. Increased inactivity may be an expression of possible behavioral problems when alongside other behaviors such as stereotypic behavior, e.g., pacing, caused by a lack of sufficient stimulation, or if it borders on lethargic behavior, which might indicate sickness or a welfare compromise when associated with low behavioral diversity [26,31,41,42,49]. Here, an ML model trained to analyze activity and inactivity may aid by giving notice when inactivity is excessively expressed, after which manual observations of more advanced behaviors, e.g., stereotypies, may be conducted to aid in a welfare assessment. However, inactivity might not in itself present a negative condition, as the jaguar could simply be content and resting rather than bored because of a lack of stimuli [31]. This is also illustrated by large felids and carnivores spending a substantial amount of time relaxing within a day [82,83,84,85].

Generally, the large amount of observed inactivity in captive jaguars may also possibly be explained by the low enclosure complexity, small enclosure size, and readily available food that favors the occurrence of inactivity compared to conditions in wild jaguar habitats [30,41]. Another possible reason for the increased inactivity in the jaguars could be explained by the small sample size or the footage being recorded continuously in their enclosure throughout the 24 h of the day, with one camera continuously filming their stable where they tend to rest.

### 4.3. Daily Activity Patterns

Another usage of activity assessments may be to assess the daily activity patterns [35,44]. The jaguars appeared to have a relatively even expression of active behavior throughout the day, although with a large amount of activity around noon from the 11th to the 13th hour (Figure 5). A study by Moreno et al. (2025) [86] measured the average movement speed across the 24 h of the day for five wild jaguars. They found that one male and one female showed the typical bimodal predator activity pattern of being less active in the daytime and more active around dusk, dawn, and at night, while three females moved more in the daytime [86]. A similar study that estimated the activity level throughout the 24 h of the day for wild jaguars found that the jaguars were mostly nocturnal and crepuscular, with variations between individuals [35]. When comparing these studies to our results, it seems that none of the captive jaguars in this proof-of-concept study had a natural bimodal nocturnal or crepuscular hunter activity pattern on the analyzed day, as they were fairly active during the daytime, with some exceptions of evening and nightly activity. For example, the mother, Chica, and the cub, Sibba, were seen walking around together outside in the 24th hour (Figure 5), which suggests that the jaguars were nocturnal to some extent. Furthermore, the jaguars might have been crepuscular to some extent, as they were observed to be active outside around dusk in the 17th hour. In addition, the jaguars were manually observed on the footage from all six recorded days to be moving around or eating outside around dusk and dawn on four different days and at night on three of those days. On two of the six recorded days, they were not observed outside at night, dusk, or dawn—only in the daytime. It could therefore be interesting for future studies to examine how often and for how long captive jaguars exhibit crepuscular and nocturnal behavior across a longer observation period.

Similar patterns of captive animals exhibiting midday activity peaks have also been observed in captive jaguars in other studies [41,43] and partly in a study of captive ocelots (*Leopardus pardalis*), who are also nocturnal hunters like jaguars [44]. Wild nocturnal hunters’ activity peaks reflect the activity peaks of their main prey [44,87], but as the jaguars in this study were fed around 13:00–13:30 in the daytime, they had no need to “hunt” at night. Another possible influence on the activity patterns of the jaguars may be the human presence, because captive animals often synchronize with human routines. A study on captive animals observed increased activity when zookeepers were present, and a possible reason may be that a caretaker can be a cue for a forthcoming event [88].

Additionally, there is only one day of analyzed footage which means that any prolonged occlusion of a jaguar will have a great effect on the daily activity pattern. Furthermore, the jaguars’ 24 h activity patterns may vary for each day, which could be examined if more days were analyzed. Despite this small sample size, the ML model still showed promise in the analysis of daily activity patterns, and a similar ML model may prove applicable in future studies.

### 4.4. Performance of the Individual Recognition

Occasional identity switching was observed, despite the model achieving a high detection mAP and high categorization F1-scores (Table 3). The identity switch mostly occurred between the two wild-type coated jaguars, Bentor and Sibba, specifically at times where they would be exhibiting similar body positions and outlines. The identity switch could be because the detector had been trained on annotations of a specific body position on only one individual, and thereby, it might confuse other individuals in this position as the one it had been trained on. Jaguars have pelage patterns that can be used for individual recognition [20,69], and it was observed that the ML model could observe such patterns. However, an ML model trained mostly on outline recognition might make a similar outline take precedence in individual recognition. Here, other studies have found success with increasing accuracy by utilizing body-part-based individual recognition, for example, the ears of wild Asian elephants (*Elephas maximus*) [20], the side of the body of captive Amur tigers (*Panthera tigris altaica*), wild Amur leopards (*Panthera pardus orientalis*), and feral cats (*Felis catus*) [89,90]. This could be a possibility for optimizing a detector for individual recognition of the studied jaguars. However, the method may require that one initially tests whether body-part-based individual recognition works with the studied species, since for some species, such as the African wild dog (*Lycaon pictus*), the method can give worse results than when utilizing the full body outline of an animal [91].

### 4.5. Possibilities in Future Research

Prior to this study, we attempted to categorize and investigate more advanced behaviors. Specifically, the captive jaguars were manually observed to be social, e.g., exhibiting allogrooming, play, and cuddling. However, the categorizer did not achieve satisfying performance based on its evaluation metrics, most likely because of similar outlines. Here, supplementing an outline-based model with body-part-based models may possibly increase the accuracy of categorization of advanced behaviors. Thus, training an ML model to classify more advanced behaviors may enable more detailed monitoring that can be used more directly in a welfare assessment, rather than merely notifying when inactivity is excessively expressed. For example, a study by Chen et al. (2026) performed automated behavioral analysis with classification of more advanced behaviors (e.g., feeding and stereotypical pacing) using an ML model on footage of a captive Amur tiger (*P. tigris altaica*) [60].

Moreover, prior to this study, it was also attempted to automatically track the activity of wild jaguars on a small sample of footage from camera traps in Bigai, which is a nature reserve in Ecuador that has been preserved since 2012 [92]. The purpose of this was to infer the behavior of wild conspecifics using an ML model trained only on footage of the three captive jaguars in Randers Regnskov. The reasoning was that this method might be applicable when studying wild animals for conservation purposes, as collecting the large amount of footage needed for training ML models may be more difficult with wild animals than with captive ones. For example, wild animals may have low population densities and be difficult to localize [3,15,19]. The ML model showed promise with detection and activity tracking, although it had some difficulty (e.g., detecting vegetation as a jaguar), seemingly caused by the novel environment. Thus, the method may possibly be enhanced when supplemented with footage from camera traps in a wild habitat. This method could possibly aid in the automatic monitoring of wild animals to elucidate their behavioral patterns, as this is an important aspect of wildlife conservation [13,14], and we therefore encourage more research on this matter.

## 5. Conclusions

The ML model developed with LabGym demonstrated the ability to detect the jaguars in Randers Regnskov and determine their space use patterns. Clear repeated movement tracks in the outdoor enclosure were visible on the heatmaps, suggesting locations for possible patrolling routes. Moreover, the ML model showed promise with individual recognition, although it struggled with occasional identity switching. Furthermore, based on the activity tracked automatically throughout the day by the ML model, the jaguars did not seem to exhibit natural bimodal nocturnal or crepuscular hunter activity patterns. Additionally, the jaguars were significantly more inactive than active on the analyzed day. Overall, the use of ML methods for individual recognition, activity tracking, and space use monitoring have shown to be a promising tool for monitoring captive jaguars in this proof-of-concept study.

## Figures and Tables

**Table 1:** Summary of information regarding the three captive jaguars (*Panthera onca*) in Randers Regnskov, Tropical Zoo (Randers Regnskov), Denmark.

Name	Sex	Age at Time of Study	Coat Type	Born	Time at Randers Regnskov (Years)	Parents
Chica	Female	11 years	Melanistic	Skærup Zoo in Denmark in 2014	11	-
Bentor	Male	5 years	Wild-type	Loro Parque in Tenerife in 2019	4	-
Sibba	Female	13 months	Wild-type	Randers Regnskov in Denmark in 2024	1	Chica and Bentor

**Table 2:** Behavioral ethogram applied in this study for jaguars in captivity in Randers Regnskov, Tropical Zoo.

Behavioral Categories	Definition
Active	Jaguar has major movements of either the entire body or one or more individual body parts, including behaviors such as mating, running, jumping, walking, swimming, playing, and pacing.
Inactive	Jaguar can be lying, sitting, or standing with no major movements of individual body parts, including minor postural adjustments such as head tilting or weight shifts.

**Table 3:** Summary of the development and use of the detector and categorizer, including evaluation metrics: (**a**) number and percentage of training and testing frames for the detector as well as its mean average precision (mAP); (**b**) number of training examples for the categorizer as well as its precision, recall, and F1-score for each behavior categorization.

**(a)**											
**Detector**			**Training frames**			**Testing frames**			**mAP**		
Identifies individual jaguars			3091 (80%)			773 (20%)			83.96%		
**(b)**											
**Categorizer**				**Number of training examples**				**Precision, recall, and F1-score**			
Active and inactive behavior				Active: 1157 Inactive: 943				Active: 0.92 Inactive: 0.90			

## Data Availability

The data presented in this study are available on request from the corresponding author. The reason for this is that the data include sensitive and private material, specifically video footage from a zoological institution and from camera traps in Bigai. Due to ethical considerations and agreements with Randers Regnskov, Tropical Zoo, the data cannot be shared publicly.

## Abbreviations

The following abbreviations are used in this manuscript:

ML	Machine learning
GUI	Graphical user interface
Randers Regnskov	Randers Regnskov, Tropical Zoo
DNN	Deep neural network
mAP	Mean average precision

## References

[1] Weckel M., Giuliano W., Silver S. (2006). Cockscomb Revisited: Jaguar Diet in the Cockscomb Basin Wildlife Sanctuary, Belize. Biotropica.

[2] Weckel M., Giuliano W., Silver S. (2006). Jaguar (*Panthera onca*) Feeding Ecology: Distribution of Predator and Prey through Time and Space. J. Zool..

[3] Blake J.G., Mosquera D., Guerra J., Loiselle B.A., Romo D., Swing K. (2014). Yasuní—A Hotspot for Jaguars *Panthera onca* (Carnivora: Felidae)? Camera-Traps and Jaguar Activity at Tiputini Biodiversity Station, Ecuador. Rev. Biol. Trop..

[4] Hayward M.W., Kamler J.F., Montgomery R.A., Newlove A., Rostro-García S., Sales L.P., Van Valkenburgh B. (2016). Prey Preferences of the Jaguar *Panthera onca* Reflect the Post-Pleistocene Demise of Large Prey. Front. Ecol. Evol..

[5] De la Torre J.A., González-Maya J.F., Zarza H., Ceballos G., Medellín R.A. (2018). The Jaguar’s Spots Are Darker than They Appear: Assessing the Global Conservation Status of the Jaguar *Panthera onca*. Oryx.

[6] Quigley H., Foster R., Petracca L., Payan E., Salom R., Harmsen B. *Panthera onca* (Errata Version Published in 2018). The IUCN Red List of Threatened Species 2017: E.T15953A123791436. https://dx.doi.org/10.2305/IUCN.UK.2017-3.RLTS.T15953A50658693.en.

[7] Rabinowitz A., Zeller K.A. (2010). A Range-Wide Model of Landscape Connectivity and Conservation for the Jaguar, *Panthera onca*. Biol. Conserv..

[8] Ripple W.J., Estes J.A., Beschta R.L., Wilmers C.C., Ritchie E.G., Hebblewhite M., Berger J., Elmhagen B., Letnic M., Nelson M.P. (2014). Status and Ecological Effects of the World’s Largest Carnivores. Science.

[9] Mena J.L., Yagui H., Tejeda V., Cabrera J., Pacheco-Esquivel J., Rivero J., Pastor P. (2020). Abundance of Jaguars and Occupancy of Medium- and Large-Sized Vertebrates in a Transboundary Conservation Landscape in the Northwestern Amazon. Glob. Ecol. Conserv..

[10] Thompson J.J., Morato R.G., Niebuhr B.B., Alegre V.B., Oshima J.E.F., de Barros A.E., Paviolo A., de la Torre J.A., Lima F., McBride R.T. (2021). Environmental and Anthropogenic Factors Synergistically Affect Space Use of Jaguars. Curr. Biol..

[11] Espinosa S., Celis G., Branch L.C. (2018). When Roads Appear Jaguars Decline: Increased Access to an Amazonian Wilderness Area Reduces Potential for Jaguar Conservation. PLoS ONE.

[12] Gaitán C.A., González-Castillo V.R., Guzmán-Flores G.D., Aguilera A.L., García M.J. (2021). Visitation Patterns of Jaguars *Panthera onca* (Carnivora: Felidae) to Isolated Water Ponds in a Tropical Forest Landscape. Therya.

[13] Festa-Bianchet M., Apollonio M. (2003). Animal Behavior and Wildlife Conservation.

[14] Macdonald D.W. (2016). Animal Behaviour and Its Role in Carnivore Conservation: Examples of Seven Deadly Threats. Anim. Behav..

[15] Archard G.A., Braithwaite V.A. (2010). The Importance of Wild Populations in Studies of Animal Temperament. J. Zool..

[16] Greggor A.L., Berger-Tal O., Blumstein D.T., Angeloni L., Bessa-Gomes C., Blackwell B.F., St Clair C.C., Crooks K., de Silva S., Fernández-Juricic E. (2016). Research Priorities from Animal Behaviour for Maximising Conservation Progress. Trends Ecol. Evol..

[17] Greggor A.L., Blumstein D.T., Wong B.B.M., Berger-Tal O. (2019). Using Animal Behavior in Conservation Management: A Series of Systematic Reviews and Maps. Environ. Evid..

[18] Rahman T., Candolin U. (2022). Linking Animal Behavior to Ecosystem Change in Disturbed Environments. Front. Ecol. Evol..

[19] Beaulieu M. (2024). Capturing Wild Animal Welfare: A Physiological Perspective. Biol. Rev..

[20] Silver S.C., Ostro L.E.T., Marsh L.K., Maffei L., Noss A.J., Kelly M.J., Wallace R.B., Gómez H., Ayala G. (2004). The Use of Camera Traps for Estimating Jaguar *Panthera onca* Abundance and Density Using Capture/Recapture Analysis. Oryx.

[21] Nakashima Y., Fukasawa K., Samejima H. (2018). Estimating Animal Density without Individual Recognition Using Information Derivable Exclusively from Camera Traps. J. Appl. Ecol..

[22] Cerqueira R.C., de Rivera O.R., Jaeger J.A.G., Grilo C. (2021). Direct and Indirect Effects of Roads on Space Use by Jaguars in Brazil. Sci. Rep..

[23] Morato R.G., Thompson J.J., Paviolo A., de La Torre J.A., Lima F., McBride R.T., Paula R.C., Cullen L., Silveira L., Kantek D.L.Z. (2018). Jaguar Movement Database: A GPS-Based Movement Dataset of an Apex Predator in the Neotropics. Ecology.

[24] Schaller G.B., Crawshaw P.G. (1980). Movement Patterns of Jaguar. Biotropica.

[25] Palagi E., Bergman T.J. (2021). Bridging Captive and Wild Studies: Behavioral Plasticity and Social Complexity in *Theropithecus gelada*. Animals.

[26] Campbell-Ward M. (2023). Quality-of-Life Assessments in Zoo Animals: Not Just for the Aged and Charismatic. Animals.

[27] Bacon H. (2018). Behaviour-Based Husbandry—A Holistic Approach to the Management of Abnormal Repetitive Behaviors. Animals.

[28] Sherwen S.L., Hemsworth L.M., Beausoleil N.J., Embury A., Mellor D.J. (2018). An Animal Welfare Risk Assessment Process for Zoos. Animals.

[29] Wolfensohn S., Shotton J., Bowley H., Davies S., Thompson S., Justice W.S.M. (2018). Assessment of Welfare in Zoo Animals: Towards Optimum Quality of Life. Animals.

[30] De Azevedo C.S., Cipreste C.F., Pizzutto C.S., Young R.J. (2023). Review of the Effects of Enclosure Complexity and Design on the Behaviour and Physiology of Zoo Animals. Animals.

[31] Tallo-Parra O., Salas M., Manteca X. (2023). Zoo Animal Welfare Assessment: Where Do We Stand?. Animals.

[32] Rabinowitz A.R., Nottingham B.G. (1986). Ecology and Behaviour of the Jaguar (*Panthers onca*) in Belize, Central America. J. Zool..

[33] Sellinger R.L., Ha J.C. (2005). The Effects of Visitor Density and Intensity on the Behavior of Two Captive Jaguars (*Panthera onca*). J. Appl. Anim. Welf. Sci..

[34] Morato R.G., Stabach J.A., Fleming C.H., Calabrese J.M., De Paula R.C., Ferraz K.M.P.M., Kantek D.L.Z., Miyazaki S.S., Pereira T.D.C., Araujo G.R. (2016). Space Use and Movement of a Neotropical Top Predator: The Endangered Jaguar. PLoS ONE.

[35] Jędrzejewski W., Vivas I., Abarca M., Lampo M., Morales L.G., Gamarra G., Schmidt K., Hoogesteijn R., Carreño R., Puerto M.F. (2021). Effect of Sex, Age, and Reproductive Status on Daily Activity Levels and Activity Patterns in Jaguars (*Panthera onca*). Mamm. Res..

[36] Price E.E., Stoinski T.S. (2007). Group Size: Determinants in the Wild and Implications for the Captive Housing of Wild Mammals in Zoos. Appl. Anim. Behav. Sci..

[37] DiVincenti L., McDowell A., Herrelko E.S. (2023). Integrating Individual Animal and Population Welfare in Zoos and Aquariums. Animals.

[38] Cavalcanti S.M.C., Gese E.M. (2009). Spatial Ecology and Social Interactions of Jaguars (*Panthera onca*) in the Southern Pantanal, Brazil. J. Mammal..

[39] Jędrzejewski W., Hoogesteijn R., Devlin A.L., Tortato F., Concone H.V.B., Azevedo F., Eriksson C.E., Fragoso C.E., Abarca M., Morato R.G. (2022). Collaborative Behaviour and Coalitions in Male Jaguars (*Panthera onca*)—Evidence and Comparison with Other Felids. Behav. Ecol. Sociobiol..

[40] Mason G.J. (1991). Stereotypies and Suffering. Behav. Process..

[41] Boccacino D., Maia C.M., Dos Santos E.F., Santori R.T. (2020). Inactivity at Night: A Case Study of the Nocturnal Behaviors of Two Captive *Panthera onca* (Felidae) Specimens. Acta biol. Colomb..

[42] Hart B.L. (1988). Biological Basis of the Behavior of Sick Animals. Neurosci. Biobehav. Rev..

[43] Boccacino D., Maia C.M., Santos E.F.D., Santori R.T. (2018). Effects of Environmental Enrichments on the Behaviors of Four Captive Jaguars: Individuality Matters. Oecol. Aust..

[44] Weller S.H., Bennett C.L. (2001). Twenty-Four Hour Activity Budgets and Patterns of Behavior in Captive Ocelots (*Leopardus pardalis*). Appl. Anim. Behav. Sci..

[45] Rees P.A. (2009). Activity Budgets and the Relationship between Feeding and Stereotypic Behaviors in Asian Elephants (*Elephas maximus*) in a Zoo. Zoo Biol..

[46] Auer U., Kelemen Z., Engl V., Jenner F. (2021). Activity Time Budgets—A Potential Tool to Monitor Equine Welfare?. Animals.

[47] Yon L., Williams E., Harvey N.D., Asher L. (2019). Development of a Behavioural Welfare Assessment Tool for Routine Use with Captive Elephants. PLoS ONE.

[48] Foster V.C., Sarmento P., Sollmann R., Tôrres N., Jácomo A.T.A., Negrões N., Fonseca C., Silveira L. (2013). Jaguar and Puma Activity Patterns and Predator-Prey Interactions in Four Brazilian Biomes. Biotropica.

[49] Miller L.J., Vicino G.A., Sheftel J., Lauderdale L.K. (2020). Behavioral Diversity as a Potential Indicator of Positive Animal Welfare. Animals.

[50] Drea C.M., Hawk J.E., Glickman S.E. (1996). Aggression Decreases as Play Emerges in Infant Spotted Hyaenas: Preparation for Joining the Clan. Anim. Behav..

[51] Stagkourakis S., Williams P., Spigolon G., Khanal S., Ziegler K., Heikkinen L., Fisone G., Broberger C. (2024). Maternal Aggression Driven by the Transient Mobilisation of a Dormant Hormone-Sensitive Circuit. bioRxiv.

[52] Mellor D.J., Beausoleil N.J. (2015). Extending the ‘Five Domains’ Model for Animal Welfare Assessment to Incorporate Positive Welfare States. Anim. Welf..

[53] Lund S.M., Nielsen J., Gammelgård F., Nielsen M.G., Jensen T.H., Pertoldi C. (2024). Behavioral Coding of Captive African Elephants (*Loxodonta africana*): Utilizing DeepLabCut and Create ML for Nocturnal Activity Tracking. Animals.

[54] Modena P.Z., Adania C.H., Lopez V.M., Guillermo-Ferreira R. (2023). Maternal Behavioural Analysis during a Successful Captive Breeding of Jaguars *Panthera onca*. Theriogenology Wild.

[55] Suárez P., Recuerda P., Arias-de-Reyna L. (2017). Behaviour and Welfare: The Visitor Effect in Captive Felids. Anim. Welf..

[56] Nielsen J., Gammelgård F., Lund S.M., Præstekær A.S.B., Frandsen A.V., Strandqvist C., Nielsen M.H., Olsen R.N., Pagh S., Faddersbøll T.L. (2026). Olfactory Enrichment of Captive Pygmy Hippopotamuses with Applied Machine Learning. Animals.

[57] Rast W., Kimmig S.E., Giese L., Berger A. (2020). Machine Learning Goes Wild: Using Data from Captive Individuals to Infer Wildlife Behaviours. PLoS ONE.

[58] Hardin A., Schlupp I. (2022). Using Machine Learning and DeepLabCut in Animal Behavior. Acta Ethol..

[59] Hu Y., Ferrario C.R., Maitland A.D., Ionides R.B., Ghimire A., Watson B., Iwasaki K., White H., Xi Y., Zhou J. (2023). *LabGym*: Quantification of User-Defined Animal Behaviors Using Learning-Based Holistic Assessment. Cell Rep. Methods.

[60] Chen L.-D., Dodds S., McGuire M., Franke M., Mastromonaco G. (2026). PantherAI: An Autonomous Behavioural Monitoring Tool for Assessing Activity Budget and Space Use in a Zoo-Housed Tiger. Ecol. Inform..

[61] Margulis S.W., Westhus E.J. (2008). Evaluation of Different Observational Sampling Regimes for Use in Zoological Parks. Appl. Anim. Behav. Sci..

[62] Altmann J. (1974). Observational Study of Behavior: Sampling Methods. Behaviour.

[63] Lund S.M., Gammelgård F., Nielsen J., Larsen L.L.N., Christensen N., Hansen S.P., Kristensen T., Rodkjær H.H.Ø., Sivagnanasundram S.M., Thomsen B.Ø. (2025). Comparing Manual and Automated Spatial Tracking of Captive Spider Monkeys Using Heatmaps. Animals.

[64] Ardoin T., Sueur C. (2024). Automatic Identification of Stone-Handling Behaviour in Japanese Macaques Using LabGym Artificial Intelligence. Primates.

[65] Hu Y. The Extended User Guide for LabGym (v2.5.1). https://github.com/yujiahu415/LabGym/blob/master/LabGym_extended_user_guide.pdf.

[66] Microsoft Corporation Microsoft Word.

[67] RectLabel. https://rectlabel.com/.

[68] Roboflow.

[69] Nipko R.B., Holcombe B.E., Kelly M.J. (2020). Identifying Individual Jaguars and Ocelots via Pattern-Recognition Software: Comparing HotSpotter and Wild-ID. Wildl. Soc. Bull..

[70] *Posit Team RStudio: Integrated Development Environment for R*; Posit Software, PBC: Boston, MA, USA. https://posit.co/.

[71] Wickham H., François R., Henry L., Müller K., Vaughan D. (2023). Dplyr: A Grammar of Data Manipulation.

[72] Wickham H., Chang W., Henry L., Pedersen T.L., Takahashi K., Wilke C., Woo K., Yutani H., Dunnington D., van den Brand T. (2016). Ggplot2: Elegant Graphics for Data Analysis.

[73] Wickham H., Henry L. (2023). Purrr: Functional Programming Tools.

[74] Wickham H., Averick M., Bryan J., Chang W., McGowan L.D., François R., Grolemund G., Hayes A., Henry L., Hester J. (2019). Welcome to the Tidyverse. J. Open Source Softw..

[75] Wickham H. (2023). Stringr: Simple, Consistent Wrappers for Common String Operations.

[76] Ooms J. (2024). Writexl: Export Data Frames to Excel “xlsx” Format.

[77] OpenAI (GPT-5, GPT-5 Mini, GPT-5.1, GPT-5.1 Mini). https://chatgpt.com.

[78] Microsoft Corporation Microsoft Excel.

[79] Zuerl M., Stoll P., Brehm I., Sueskind J., Raab R., Petermann J., Zanca D., Simon R., von Fersen L., Eskofier B. (2024). Automated Long-Term Monitoring of Stereotypical Movement in Polar Bears under Human Care Using Machine Learning. Ecol. Inform..

[80] Maulana R., Gawi J.M., Utomo S.W. (2020). Architectural Design Assessment of Javan Leopard Rehabilitation Facility Regarding the Occurrence of Stereotypical Pacing. IOP Conf. Ser. Earth Environ. Sci..

[81] Giese L., Melzheimer J., Bockmühl D., Wasiolka B., Rast W., Berger A., Wachter B. (2021). Using Machine Learning for Remote Behaviour Classification—Verifying Acceleration Data to Infer Feeding Events in Free-Ranging Cheetahs. Sensors.

[82] Bryce C.M., Dunford C.E., Pagano A.M., Wang Y., Borg B.L., Arthur S.M., Williams T.M. (2022). Environmental Correlates of Activity and Energetics in a Wide-Ranging Social Carnivore. Anim. Biotelemetry.

[83] Cozzi G., Broekhuis F., McNutt J.W., Turnbull L.A., Macdonald D.W., Schmid B. (2012). Fear of the Dark or Dinner by Moonlight? Reduced Temporal Partitioning among Africa’s Large Carnivores. Ecology.

[84] Siegel J.M. (2005). Clues to the Functions of Mammalian Sleep. Nature.

[85] Seyrling I., Dierkes P.W., Burger A.L. (2022). Diurnal and Nocturnal Behaviour of Cheetahs (*Acinonyx jubatus*) and Lions (*Panthera leo*) in Zoos. Animals.

[86] Moreno R., de la Torre J.A., Ortega J., Young N., Puertes A., Kays R. (2025). Jaguar Space Use in the Working Landscape of Darien, Panama. Trop. Conserv. Sci..

[87] Carrillo E., Fuller T.K., Saenz J.C. (2009). Jaguar (*Panthera onca*) Hunting Activity: Effects of Prey Distribution and Availability. J. Trop. Ecol..

[88] Pacheco E., Krebs B.L., Watters J.V. (2024). Keeper Effect: Animals Are More Active in the Presence of Their Caretakers. Zoo Biol..

[89] Shi C., Xu J., Roberts N.J., Liu D., Jiang G. (2023). Individual Automatic Detection and Identification of Big Cats with the Combination of Different Body Parts. Integr. Zool..

[90] Akbar R.R.S., Rees M.W., Fleming P.A., Sohel F. (2025). Body-Part-Based Individual Feral Cat Identification from Camera Trap Images Using Deep Learning. Ecol. Inform..

[91] De Lorm T.A., Horswill C., Rabaiotti D., Ewers R.M., Groom R.J., Watermeyer J., Woodroffe R. (2023). Optimizing the Automated Recognition of Individual Animals to Support Population Monitoring. Ecol. Evol..

[92] Bigai: Randers Regnskov—Tropical Zoo. https://www.regnskoven.dk/laerbevar/naturbevarelse/bigai/.

